# Effects of three types of resistance training on knee osteoarthritis: A systematic review and network meta-analysis

**DOI:** 10.1371/journal.pone.0309950

**Published:** 2024-12-05

**Authors:** Yutao Jiang, Yajun Tan, Liang Cheng, Jintao Wang

**Affiliations:** 1 BAYI Orthopedic Hospital, China RongTong Medical Healthcare Group Co. Ltd, Chengdu, China; 2 Sport Hospital Attached To Chengdu Sport University, Chengdu, China; 3 Sichuan Sports College, Chengdu, China; 4 Sichuan Orthopedic Hospital, Chengdu, China; Nove de Julho University, BRAZIL

## Abstract

**Background:**

Resistance training (RT) is recognized in clinical guidelines as a beneficial treatment for knee osteoarthritis (KOA), but the efficacy of different RT types is not well-established.

**Objective:**

This network meta-analysis (NMA) aimed to compare the effects of different types of RT, namely, isometric muscle strengthening (IMMS), isokinetic muscle strengthening (IKMS) and isotonic muscle strengthening (ITMS), on pain, function and quadriceps muscle strength of patients with KOA.

**Methods:**

A systematic search was conducted up to September 2023 on databases, including PubMed, Cochrane Library, EMbase, Web of Science and China National Knowledge Infrastructure. The included studies comprised randomised controlled trials (RCTs) comparing RT with conventional rehabilitation and physiotherapy or other types of RT.

**Results:**

Compared with the control group (CG) that received conventional physiotherapy, IKMS was optimal in terms of pain relief (MD = -1.33, 95% CI: -1.83 to -0.83), function (MD = -12.24, 95% CI: -17.29 to -7.19) and knee extension torque (SMD = -0.44, 95% CI: -0.74 to -0.14).

**Conclusions:**

Compared with conventional rehabilitation therapy, all three types of RT can improve pain and knee-joint function in KOA patients. IKMS demonstrated the best results among the different RT modalities.

**PROSPERO registration:**

**PROSPERO registration number:** CRD42023448579.

## 1 Introduction

Osteoarthritis is a multifactorial chronic disease, with approximately 240 million people worldwide diagnosed with the condition, predominantly Knee Osteoarthritis (KOA). This disease is a primary cause of disability among the elderly and a considerable source of societal cost [1, 2]. The risk factors for KOA include age, weight, gender, joint structural changes and occupation [3]. Individuals suffering from KOA typically experience pain, joint mobility limitations and muscle weakness [3–5]. These symptoms can affect many aspects of a patient’s daily life, including mental health and quality of life, and may increase the cost of living for the patient [6].

Currently, exercise therapy is recognized as a first-line treatment strategy in the guidelines for the diagnosis and treatment of KOA worldwide [7]. Common types of exercise therapy encompass a variety of modalities, including aerobic exercises, aquatic exercises, resistance training, and mind-body exercises, each with its unique benefits [8]. Aquatic exercises alleviate the load on joints due to body weight but may impose a certain load on the heart [9]; aerobic exercises effectively improve cardiopulmonary function and overall endurance, yet the optimal dosage requires further investigation; mind-body exercises such as Tai Chi and Yoga are beneficial for enhancing balance, flexibility, and reducing psychological stress, though the definitive efficacy remains inconclusive [10].

Resistance training (RT) is a common rehabilitative exercise modality, and it has been widely used in the treatment of various musculoskeletal disorders [8, 11]. RT offers important benefits in the treatment of patients with KOA [12–17]. It also enhances muscle strength, improves joint stability and function and alleviates joint load, which slow down the further degeneration of knee cartilage and improve pain symptoms and the quality of life for patients. Specifically, isokinetic muscle strengthening (IKMS) is considered a safe and effective form of RT for the rehabilitative treatment of various musculoskeletal diseases [18]. However, specialised equipment is required for IKMS, which is difficult to access by patients; in addition, economic factors may hinder its adoption as a treatment modality [19]. Thus, some clinicians or physiotherapists suggest patients to undertake isometric muscle strengthening (IMMS), isotonic muscle strengthening (ITMS) and other forms of exercise therapy.

Previous meta-analyses and reviews have demonstrated that exercise plays a positive role in reducing pain and improving function in patients with KOA [20–24]. Among these, RT effectively alleviates pain, enhances functionality, and boosts muscle strength. However, comparisons of the effects of RT with aerobic exercises, aquatic exercises, and other forms of exercise remain controversial. This may be due to categorizing IMMS, ITMS, and IKMS as the same type of RT, which leads to overlooking the differences among them when assessing therapeutic outcomes.

Therefore, this network meta-analysis (NMA) aimed to evaluate the effect of IMMS, IKMS and ITMS on pain, function and muscle strength of patients with KOA. By comparing the differences among these three types of RT, this study lays the groundwork for accurately assessing various exercise therapies and provides references for physical therapists and physicians. This guidance is intended to assist in making informed decisions regarding the optimal RT modality for patients with KOA.

## 2 Method

We followed the Preferred Reporting Items for Systematic Reviews and Meta-analyses guidelines in the preparation of this article (PROSPERO registration number: CRD42023448579).

### 2.1 Inclusion criteria

#### 2.1.1 Search strategy and selection criteria

Published randomised controlled trials (RCTs) with language restrictions to Chinese and English.

The study population comprised KOA patients who met the diagnostic criteria of the Chinese Medical Association Orthopedics Branch’s ‘Guidelines for the Diagnosis and Treatment of Osteoarthritis’ [25] or the revised diagnostic criteria for KOA by the American College of Rheumatology [26].

#### 2.1.2 Interventions

The treatment groups included patients groups that received IMMS, IKMS and ITMS. The control group (CG) received standard KOA treatments other than RTs, such as physical therapy, rehabilitation or pharmacotherapy. Alternatively, comparisons were conducted among the three RT indices.

#### 2.1.3 Outcomes

The visual analogue score [27, 28] (VAS) for pain assessment was the primary outcome measure; secondary outcome measures included the Western Ontario and McMaster University Osteoarthritis Index [29] (WOMAC) for functional assessment and knee-joint isokinetic peak torque strength for muscle strength assessment.

### 2.2 Exclusion criteria

The exclusion criteria encompassed those mentioned in literature, non-Chinese and non-English publications, articles for which the full text cannot be acquired or which entailed duplicate publication or substantial content overlap; documents with conspicuous data inaccuracies, incomplete or unclear data or relevant data that remained unobtainable despite following up through the author’s correspondence.

### 2.3 Literature search strategy

We conducted a computerised search of databases, including Web of Science, PubMed, Cochrane Library, EMbase and China National Knowledge Infrastructure, to collate clinical RCTs that evaluated the efficacy of various RTs in the treatment of KOA. The search spanned from the inception of each database up to September 2023 and applied the combination of MeSH and free-text terms. The search strategy encompassed the following keywords: ‘isometric contraction’, ‘isokinetic contraction’, ‘isotonic contraction’, ‘Exercise’, ‘Resistance Training’, ‘Muscle Strength’, ‘Osteoarthritis’ (Mesh), ‘knee osteoarthritides’, ‘knee osteoarthritis’, ‘osteoarthritis of knee’, ‘osteoarthritis of the knee’, etc.

### 2.4 Literature screening and data extraction

Independent literature search was independently conducted by YJ and JW. They adhered strictly to predefined inclusion and exclusion criteria for initial and secondary screening of the literature. Data extraction was also independently performed by YJ and JW, while comparison tasks were managed by CL. In cases of discrepancies or errors, consultations were held with CL and YT. Efforts were made to supplement missing data or information by contacting the corresponding authors via email or telephone.

The main data extracted for this NMA included the first author, publication year, sample size, age, gender, intervention measures, intervention duration and relevant outcome measures.

### 2.5 Quality assessment

The Cochrane Risk of Bias tool was used for quality assessment. The tool covers seven domains of assessment: random sequence generation, allocation concealment, blinding of participants and personnel, blinding of outcome assessment, incomplete outcome data, selective reporting and other biases. The risk of bias in each domain was evaluated as low, high or unclear.

### 2.6 Statistical analysis

The statistical methods used for NMA were based on a frequentist framework. A random-effects model was applied to analyse all outcome measures. The evaluation criteria comprised continuous variables. When pain and functional outcomes had consistent units, the mean difference (MD) was used as the effect size. When inconsistency was detected in the units of knee-extension-strength outcomes, the standardised MD (SMD) was used as the effect size. The corresponding 95% confidence intervals (CIs) were calculated. Review Manager 5.3 and Stata 14 were employed to assess the quality of literature and statistical analysis, respectively. Network evidence relationship diagrams were created for each outcome measure (where each node represents an intervention, the node’s size indicates the sample size for that intervention, and the thickness of lines connecting nodes denotes the number of studies comparing the respective interventions). Inconsistency tests were conducted using an inconsistency model. However, a consistency model was used for data analysis if the p-value was greater than 0.05. Node splitting was performed to assess the local inconsistency between direct and indirect comparisons. Inconsistency factors (IFs) and the 95% CI for each closed loop in the network were calculated. The if plot command in Stata was used to detect loop inconsistency. A lower limit of the 95% CI close to or including 0 indicated a high degree of agreement between direct and indirect comparison evidence. Surface under the cumulative ranking (SUCRA) was performed to rank the interventions and identify the most effective RT.

## 3 Results

### 3.1 Literature search results

**Fig 1:**
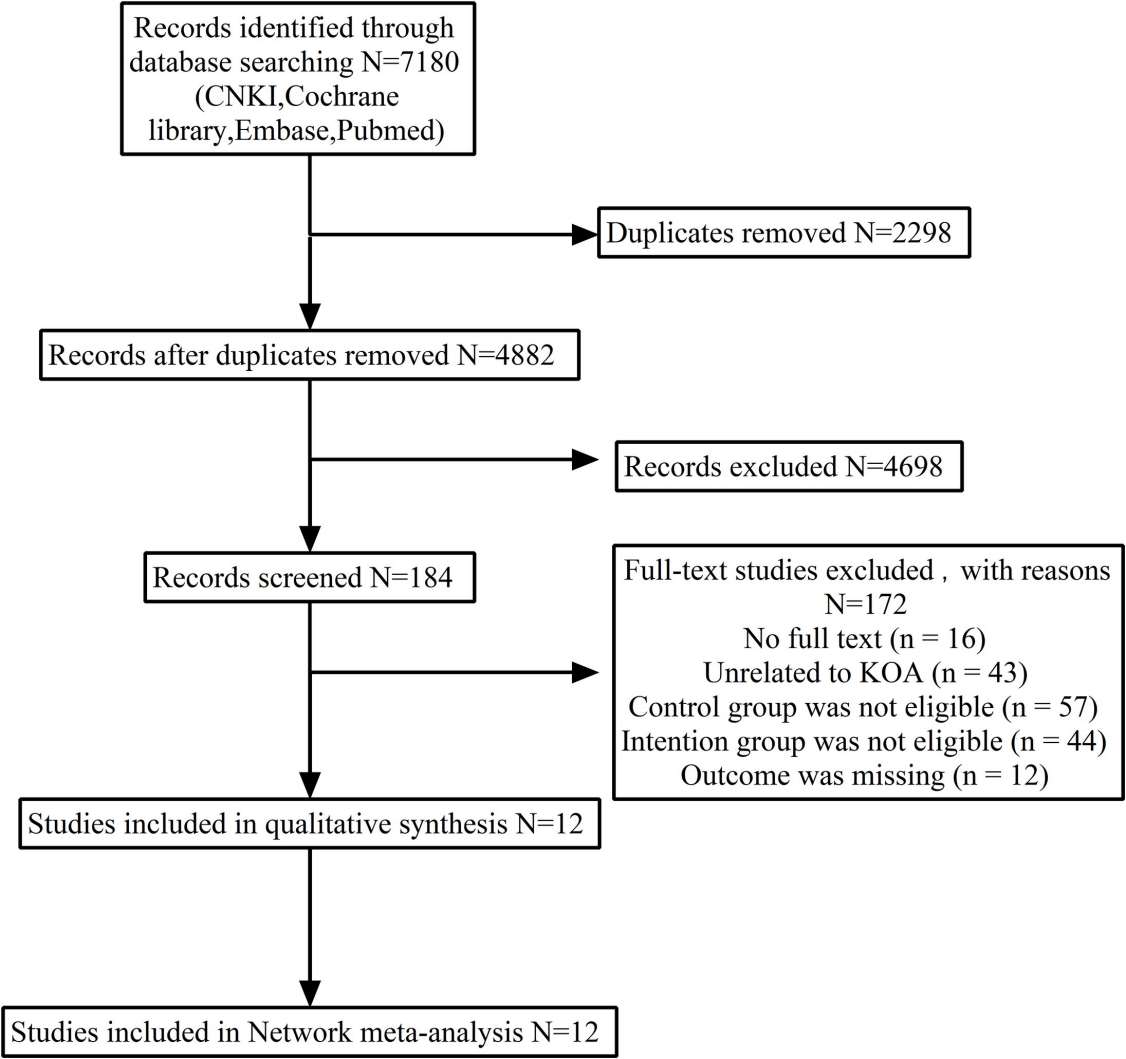
PRISMA flow diagram.

A total of 11,130 articles were obtained from the preliminary search among various sources, including China National Knowledge Infrastructure (n = 85), Web of Science (3950), PubMed (n = 3413), Embase (n = 2048) and Cochrane Library (n = 1634). After deduplication using Endnote X9 software, 4882 articles were retained. Exactly 4343 articles were kept after considering the inclusion and quality evaluation criteria. Finally, 184 articles remained after the titles and abstracts were reviewed to remove irrelevant studies. The remaining articles were further reviewed in full, which resulted in the inclusion of 12 RCTs. Fig 1 shows the specific process.

### 3.2 Characteristics of included studies

Twelve studies involving 753 patients with KOA were included in this work. The treatment duration was 3–8 weeks, and the interventions included IMMS, IKMS, ITMS and standard KOA treatments.

Table 1 presents the basic characteristics of the studies.

**Table 1:** General characters of the included study (n = 12).

Author, Year	Participants(M/F),age	Intervention	Outcome measure
1. Fevziye [38] 2013	Patients diagnosed with KOA (grade: not mentioned) n = 61(51/10) average age:58.9	Gr1: 3 weeks IMMS Gr2: 3 weeks IKMS Gr3: 3 weeks ITMS	1. VAS 2. Isokinetic peak torque at 60°/s (Peak torque/body weight)
2. Huang [39] 2003	Patients diagnosed with KOA (grade: II) n = 132(not mentioned) average age:>40	Gr1: 8 weeks IMMS Gr2: 8 weeks IKMS Gr3: 8 weeks ITMS Gr4: 8 weeks rehabilitation therapy	1. VAS 2. WOMAC
3. Bahi A [40] 2022	Patients diagnosed with KOA (grade: II or III) n = 60(4/56) average age:54.5	Gr1:3 weeks IMMS Gr2:3 weeks IKMS Gr3:3 weeks ITMS	1. VAS 2. WOMAC 3. Isokinetic peak torque at 60°/s (Peak torque)
4. Esin [30] 2018	Patients diagnosed with KOA(grade: II or III) n = 30(0/30) average age:51.9	Gr1:4 weeks IMMS Gr2:4 weeks IKMS	1. VAS 2. WOMAC
5. Salli A [31] 2010	Patients diagnosed with KOA(grade: I or II) n = 47(9/38) average age:57.0	Gr1:8 weeks IMMS Gr2:8 weeks IKMS	1. VAS 2. Isokinetic peak torque at 60°/s (Peak torque)
6. Kilinc S [32] 2019	Patients diagnosed with KOA (grade: II to IV) n = 32(0:32) average age:55.0	Gr1:4 weeks IMMS Gr2:4 weeks IKMS	1. VAS 2. Isokinetic peak torque at 60°/s (Peak torque)
7. Rang [33] 2022	Patients diagnosed with KOA (grade: not mentioned) n = 81(43:38) average age:59.1	Gr1:8 weeks IKMS Gr2:8 weeks rehabilitation and manual therapy	1. VAS
8. Ni [34] 2018	Patients diagnosed with KOA (grade: not mentioned) n = 89(not mentioned) average age:61	Gr1:6 weeks ITMS Gr2:6 weeks ultrasound and manual therapy	1. VAS 2. WOMAC
9. Anwer S [35] 2013	Patients diagnosed with KOA(grade: I to III) n = 42(not mentioned) average age:55.5	Gr1:5 weeks IMMS Gr2:5 weeks ultrasound therapy	1. WOMAC
10. Chen [36] 2020	Patients diagnosed with KOA(grade: not mentioned) n = 80(41/39) average age:56.1	Gr1:8 weeks IKMS Gr2:8 weeks rehabilitation therapy	1. VAS 2. WOMAC
11. Hou [37] 2019	Patients diagnosed with KOA (grade: I to III) n = 60(9/51) average age:56.4	Gr1:8 weeks IKMS Gr2:8 weeks manual therapy	1. WOMAC 2. Isokinetic peak torque at 60°/s (Peak torque/body weight)
12. Eyigor S [19] 2004	Patients diagnosed with KOA (grade: II to III) n = 39(6/33) average age:52.6	Gr1:6 weeks IKMS Gr2:6 weeks ITMS	1. VAS 2. WOMAC 3. Isokinetic peak torque at 60°/s (Peak torque)

Note: KOA: Knee osteoarthritis, Gr: group, IMMS: isometric muscle strengthening, IKMS: isokinetic muscle strengthening, ITMS: isotonic muscle strengthening.

### 3.3 Bias risk assessment

**Fig 2:**
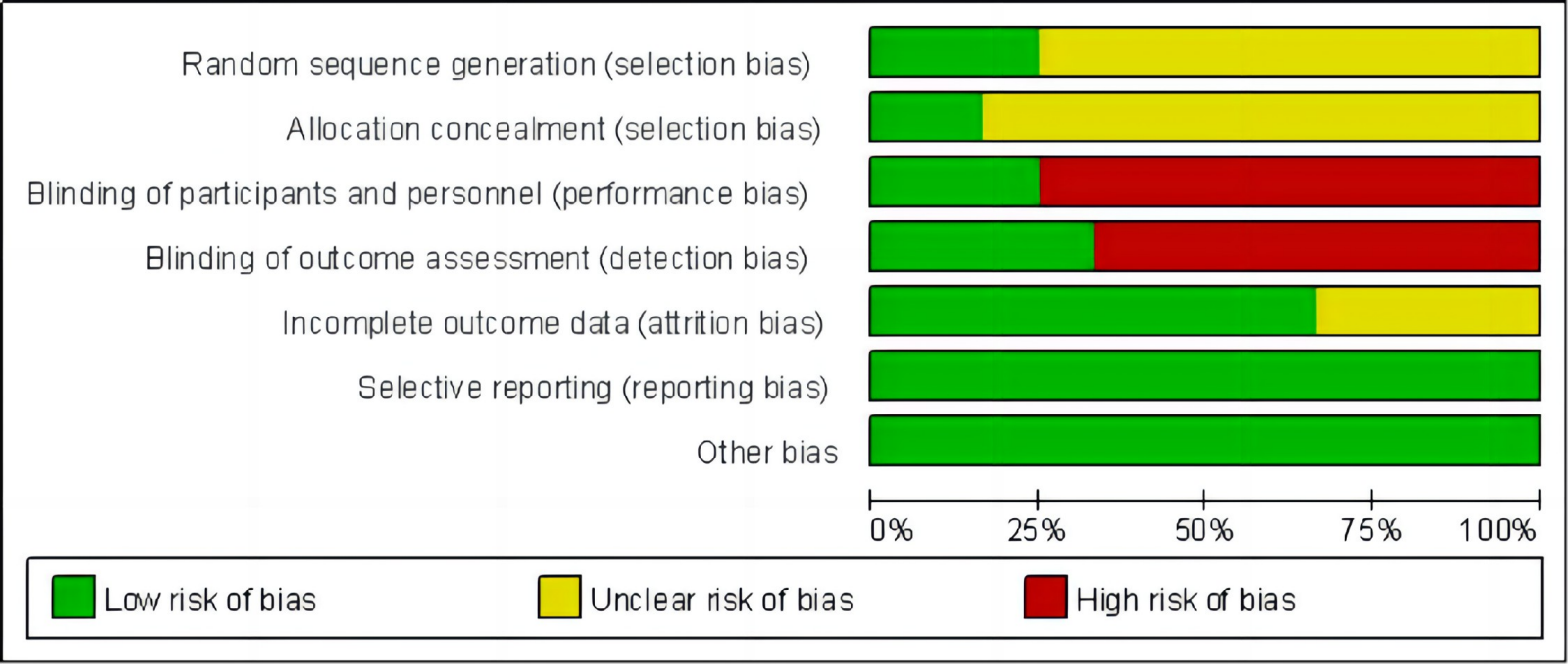
Summary of risk of bias.

**Fig 3:**
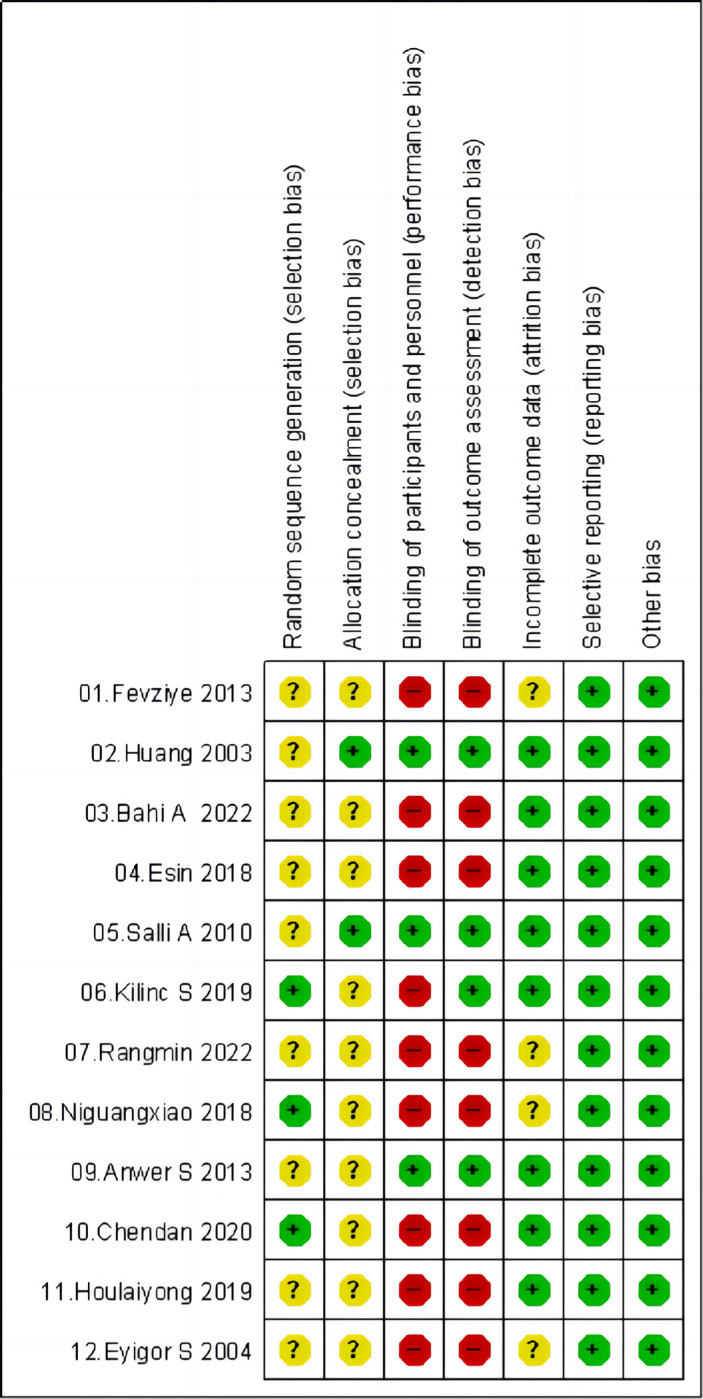
Risk of bias table.

All 12 included studies [19, 30–40] were RCTs. Among these RCTs, three papers [32, 34, 36] provided detailed descriptions of random allocation methods. Two [31, 38] mentioned the use of sealed envelopes for allocation concealment. Three research [31, 35, 38] implemented double blinding, one [31] implemented single blinding, and the rest did not specify blinding procedures. Six studies [19, 31, 33, 34, 38, 39] reported dropouts, with two [31, 38] describing the related reasons. The recruitment and outcome assessment sample sizes were the same in the remaining studies. Table 2 and Figs 2 and 3 show the bias risk.

**Table 2:** Risk of bias assessment of the included study(n = 12).

Study ID	Baseline Comparability	Random Allocation Method	Allocation Concealment	Blinding	Outcome Data Integrity	Selective Reporting of Results	Other Bias Sources
1. Fevziye	Met	Randomized control (but method not described)	Not detailed	Not blinded	Missing data, not explained	No	No
2. Huang	Met	Randomized control (but method not described)	Concealed	Double-blind	Missing data, explained	No	No
3. Bahi A	Met	Randomized control (but method not described)	Not detailed	Not blinded	Complete	No	No
4. Esin	Met	Randomized control (but method not described)	Not detailed	Not blinded	Complete	No	No
5. Salli A	Met	Randomized control (but method not described)	Concealed	Double-blind	Missing data, explained	No	No
6. Kilinc S	Met	Randomized control (Random numbers)	Not detailed	Single-blind	Complete	No	No
7. Rang	Met	Randomized control (but method not described)	Not detailed	Not blinded	Missing data, not explained	No	No
8. Ni	Met	Randomized control (Random numbers)	Not detailed	Not blinded	Missing data, not explained	No	No
9. Anwer S	Met	Randomized control (but method not described)	Not detailed	Double-blind	Complete	No	No
10. Chen	Met	Randomized control (Random numbers)	Not detailed	Not blinded	Complete	No	No
11. Hou	Met	Randomized control (but method not described)	Not detailed	Not blinded	Complete	No	No
12. Eyigor S	Met	Randomized control (but method not described)	Not detailed	Not blinded	Missing data, not explained	No	No

### 3.4 NMA

#### 3.4.1 Network diagrams

**Fig 4:**
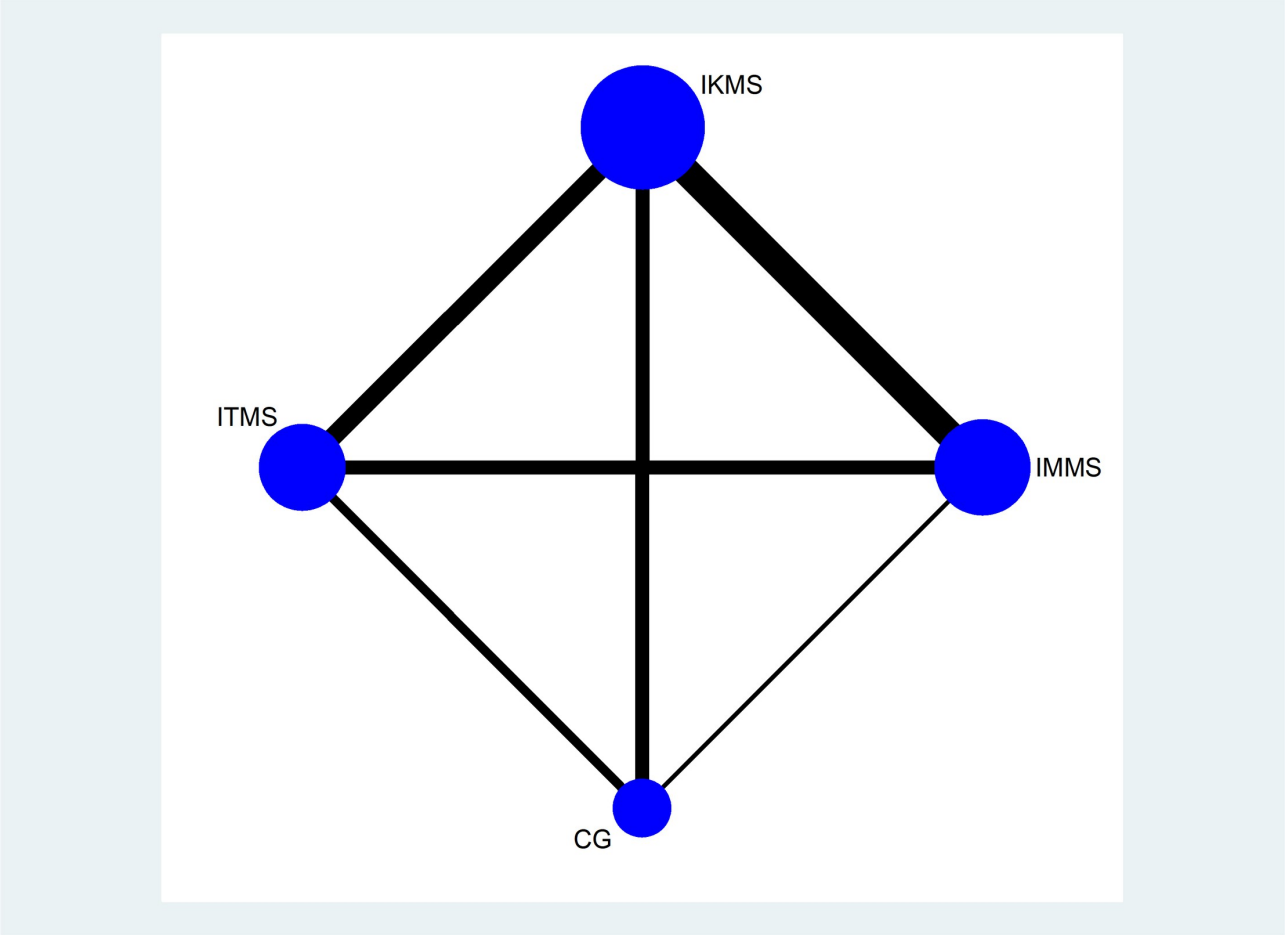
VAS network.

**Fig 5:**
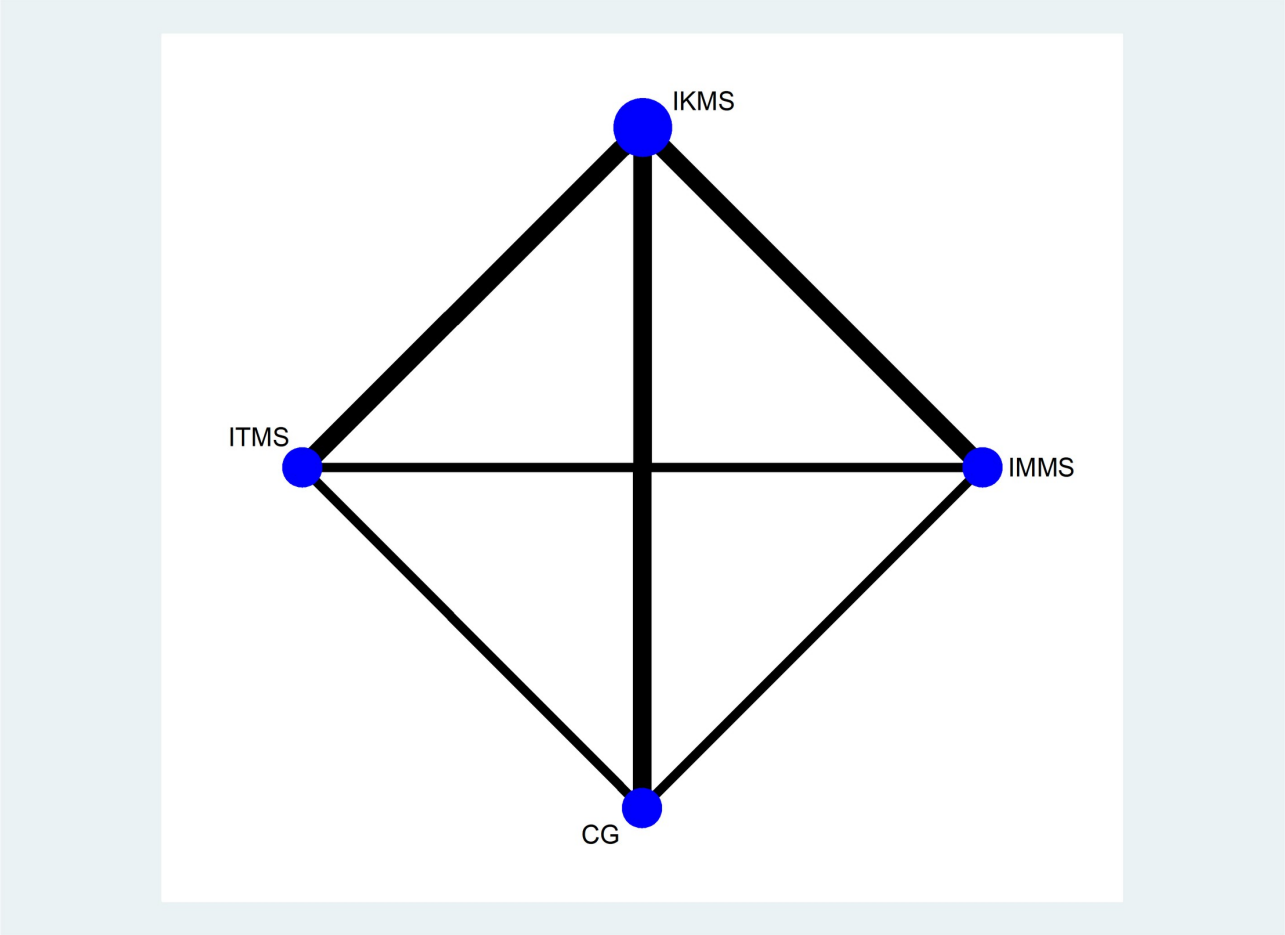
WOMAC network.

**Fig 6:**
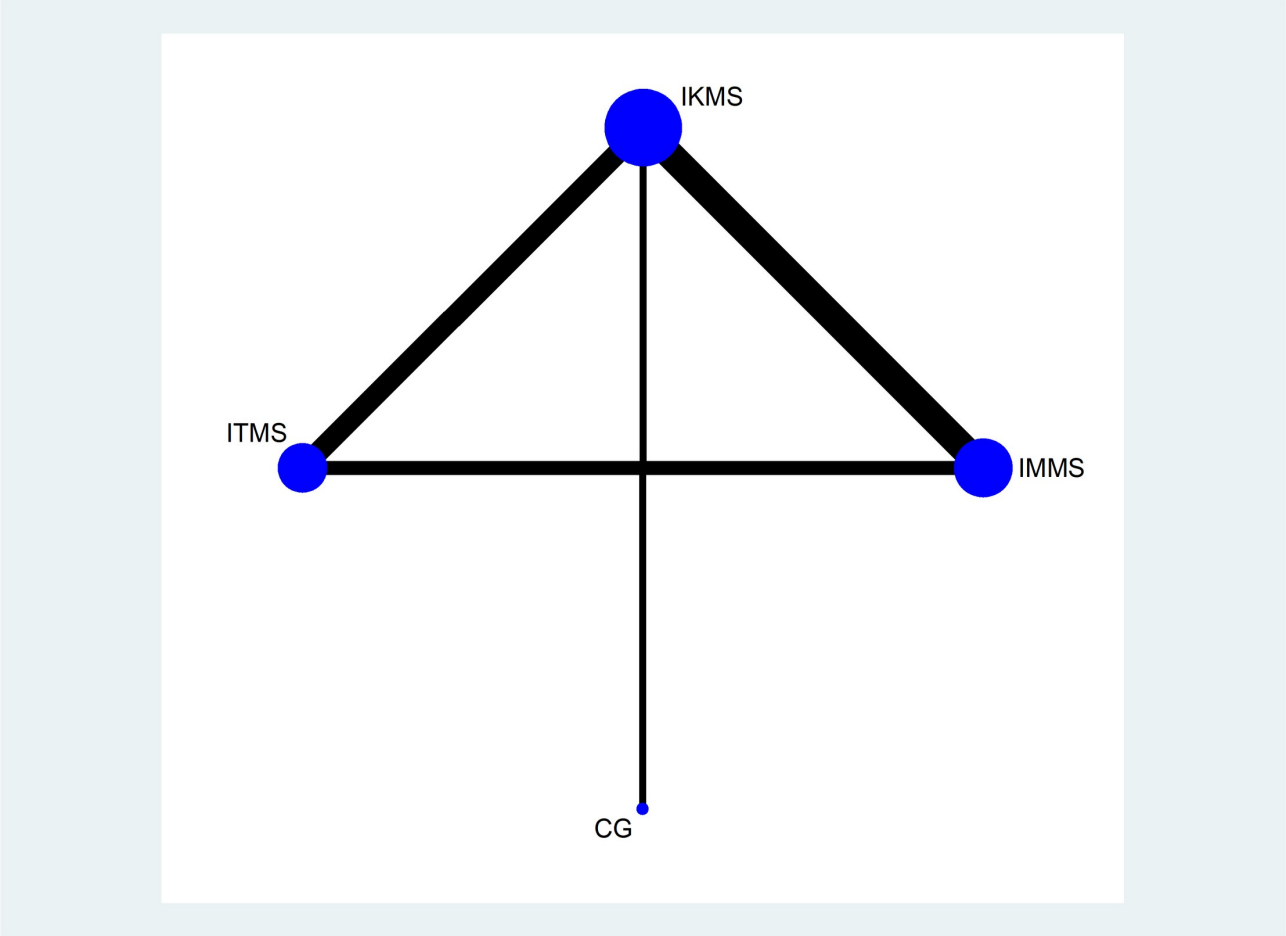
Knee-extension-torque network. Note: On the network diagrams, the size of each node is weighted according to the number of participants receiving a specific intervention, while the thickness of the lines connecting two nodes is weighted based on the number of studies applying the connected interventions. IMMS:Isometric muscle strengthening, IKMS:Isokinetic muscle strengthening, ITMS: Isotonic muscle strengthening. CG: Control Group.

Twelve articles were included, and they covered four types of interventions: IMMS, IKMS, ITMS and a CG. Figs 4–6 illustrate the network relationship.

#### 3.4.2 Consistency testing

We performed global inconsistency tests on three outcome measures. All p-values were greater than 0.05, which indicate that no statistically significant difference was observed between the direct and indirect comparisons of the studies. Thus, a consistency model was used for statistical analysis. The node-splitting method was used for local inconsistency tests, and the p-values were all greater than 0.05, which indicates no statistical significance and suggests the consistency between direct and indirect comparison results. Utilizing the Loop IFs for assessment, the 95% CI consistently demonstrated a lower limit that includes 0.

#### 3.4.3 VAS NMA results

Compared with the CG, statistically significant differences were observed among IMMS (MD = -0.74, 95% CI: -1.37 to -0.11), IKMS (MD = -1.33, 95% CI: -1.83 to -0.83) and ITMS (MD = -1.31, 95% CI: -1.90 to -0.71). Pairwise comparisons within the RT interventions revealed a statistical significance IMMS versus IKMS (MD = -0.59, 95% CI: -1.13 to -0.05). Based on SUCRA probability, the four different interventions were ranked as follows: IKMS (83.8%) > ITMS (80.9%) > IMMS (35.0%) > CG (0.4%).

Table 3 Results of NMA of the VAS of different interventions.

**Table 3:** Results of NMA of the VAS of different interventions.

Intervention	IKMS	ITMS	IMMS
ITMS	-0.02 (-0.61,0.57)	0	
IMMS	-0.59 (-1.13,-0.05)*	-0.57 (-1.23,0.09)	0
CG	-1.33 (-1.83,-0.83)*	-1.31 (-1.90,-0.71)*	-0.74 (-1.37,-0.11)*

Note: IMMS: isometric muscle strengthening, IKMS: isokinetic muscle strengthening, ITMS: isotonic muscle strengthening and CG: control group. MD > 0 indicates that the intervention in the column is better than the one in the row, MD < 0 indicates the opposite, and

* indicates statistically significant differences (P < 0.05) in the comparison between two interventions.

#### 3.4.4 WOMAC total score NMA results

Compared with the CG, IMMS (MD = -10.81, 95% CI: -16.82 to -4.80), IKMS (MD = -12.24, 95% CI: -17.29 to -7.19) and ITMS (MD = -9.00, 95% CI: -15.55 to -2.45) exhibited statistically significant differences. The other intervention comparisons revealed no statistically significant differences. Based on SUCRA probability, the four different interventions were ranked as follows: IKMS (82.9%) > IMMS (67.2%) > ITMS (49.8%) > CG (0.1%).

Table 4 NMA results on the WOMAC scores of different interventions.

**Table 4:** NMA results on the WOMAC scores of different interventions.

Intervention	IKMS	IMMS	ITMS
IMMS	-1.43 (-8.04,5.18)	0	
ITMS	-3.24 (-9.99,3.50)	-1.81 (-9.93,6.30)	0
CG	-12.24 (-17.29,-7.19)*	-10.81 (-16.82,-4.80)*	-9.00 (-15.55,-2.45)*

Note: IMMS: isometric muscle strengthening, IKMS: isokinetic muscle strengthening, ITMS: isotonic muscle strengthening and CG: control group. MD > 0 indicates that the intervention in the column is better than the one in the row, MD < 0 indicates the opposite, and

* indicates statistically significant differences (P<0.05) in the comparison between two interventions.

#### 3.4.5 60° knee-extension-torque NMA results

Compared with the CG, IKMS (SMD = -0.66, 95% CI: -1.18 to -0.14) and ITMS (SMD = -0.64, 95% CI: -1.24 to -0.04) exhibited statistically significant differences. IMMS showed statistical significance compared with IKMS (SMD = -0.44, 95% CI: -0.74 to -0.14) and ITMS (SMD = -0.42, 95% CI: -0.78 to -0.06). The remaining intervention methods showed no statistically significant differences. Based on SUCRA probability, the four different interventions were ranked as follows: IKMS (85.0%) > ITMS (80.5%) > IMMS (26.1%) > CG (8.4%).

Table 5 NMA results on the isokinetic peak torque of different interventions.

**Table 5:** NMA results on the isokinetic peak torque of different interventions.

Intervention	IKMS	ITMS	IMMS
ITMS	-0.02 (-0.33,0.28)	0	
IMMS	-0.44 (-0.74,-0.14)*	-0.42 (-0.78,-0.06)*	0
CG	-0.66 (-1.18,-0.14)*	-0.64 (-1.24,-0.04)*	-0.22 (-0.82,0.38)

Note: IMMS: isometric muscle strengthening, IKMS: isokinetic muscle strengthening, ITMS: isotonic muscle strengthening and CG: control group. SMD > 0 indicates that the intervention in the column is superior to the intervention in the row, SMD < 0 indicates that the intervention in the row is superior to the intervention in the column

* indicates statistically significant differences between the two intervention methods (P<0.05).

## 4 Discussion

This NMA recommended IKMS as the preferred frontline RT method for KOA patients, citing its superior effectiveness in comparison studies. Whether assessed using the VAS, WOMAC or knee extensor moment, IKMS emerged as the most effective method. In terms of the VAS and knee extensor moment, ITMS was superior to IMMS and inferior to IKMS. Meanwhile, in consideration of WOMAC, IMMS was inferior to IKMS but superior to ITMS. Notably, although IMMS demonstrated superiority over the CG in terms of knee extensor moment, no significant statistical difference was found between them. ITMS and IMMS have the advantage of lower cost and are easier implementation than IKMS, which render them viable RT options for clinical therapists when providing KOA patients with suitable RT modes, contingent on the situation.

In the included studies of this NMA, four studies did not mention the Kellgren-Lawrence (KL) classification of KOA, six studies involved patients with KL grade III KOA, and one study included patients with KL grade IV KOA. Research indicates that although the KL grade serves as an imaging classification for KOA, it is closely associated with symptoms such as pain and functional impairment in patients [41]. However, the other research [42] indicates that the primary factors contributing to disability in KOA are weakness of the quadriceps, pain, and age, rather than the KL grade. The discrepancy may be due to the KL grading system not differentiating between the degenerative changes in the medial and lateral compartments. Singh [43] suggests that osteophytes on the medial side of the knee, compression of the medial meniscus, and degeneration of the medial collateral ligament are more closely related to pain and functional impairment, whereas there is no significant correlation with lateral degeneration. Furthermore, studies have shown that changes in the mechanical axis related to knee instability in KOA can be effectively addressed by enhancing the strength of the quadriceps and neuromuscular adaptation, thereby improving knee stability [44, 45]. Therefore, resistance training is essential for patients with different KL grades of KOA.

These findings have clinical importance given that quadriceps muscle weakness is commonly observed in early KOA regardless of the absence of knee pain or muscle atrophy [46]. Muscle weakness is a primary cause of functional limitation in KOA patients, and strengthening the quadriceps and hamstrings helps in maintaining and increasing strength, joint stability and the range of motion while alleviating pain [47]. Thus, RT is applicable for most KOA patients. Pain is one of the most common symptoms of KOA, and it entails a complex pathophysiology [48, 49]. The pathogenesis of OA also involves metabolic changes in the joint cartilage, subchondral bone and synovium [48, 50]. These alterations affect metabolic pathways in chondrocytes, synovial cells and osteocytes, which interact with the immune system via inflammatory mediators [49]. Other pathophysiological mechanisms include neural peripheries and central abnormalities [51]^.^ Although the mechanism by which RT alleviates KOA pain remains unclear, RT can alleviate neural abnormalities, reduce pain sensitivity and attenuate nociception [52]. In addition, RT improves proprioception in peripheral muscles and knee-joint coordination [53]. According to previous meta-analyses, RT enhances peri-knee muscular strength, increases knee-joint stability, improves function and concurrently mitigates pain [54], which align with the findings of this NMA.

The NMA collected studies with training durations ranging from 3 weeks to 8 weeks. Short-term training may induce strength improvements related to neural activation and the recruitment of nerves and muscles rather than muscular hypertrophy [55]. Fevziye [38] employed an intervention involving cross-training comparison. They observed that through intervention on one side of the affected limb and leaving the other side untreated, improvements were observed not only in the pain levels but also in the muscle strength of the untreated limb. This finding proves the presence of neural adaptation. The trial also additionally included single-leg standing and a 50-meter walk time as test indicators for patients with KOA. The results demonstrated that all three types of RT improved both the static stability and walking function in patients with KOA, with IMMS showing the most significant improvements in both tests. However, in the control group, IMMS was less effective than IKMS and ITMS, indicating that IKMS and ITMS are superior in enhancing neural adaptation. The study has shown that during isokinetic training, skeletal muscles can better recruit and activate nerves, which resulted in substantial strength gains [56]. Guilhem [57] posits that eccentric contractions more effectively facilitate neuromuscular recruitment. During eccentric contractions, muscles can distribute mechanical stress more effectively, promoting neuromuscular recruitment. Additionally, Research has indicated that the abnormal torque of the quadriceps muscle caused by KOA is also associated with a decrease in neuromuscular recruitment capabilities. The reflex inhibition caused by KOA pain leads to atrophy and weakness of the vastus medialis muscle, resulting in a change in the lower limb force line of KOA and exacerbating the varus deformity of KOA [58]. Strengthening the opposing hamstring muscles in addition to the quadriceps may also improve function in KOA patients [53]. During muscle lengthening contractions, opposing muscles can also be activated, and antagonistic muscles exhibit a higher neuromuscular efficiency than agonist muscles [59], which may explain the better performance of IMMS in improving function but inferiority to ITMS in terms of strength improvement. A review has suggested the higher effectiveness of IMMS and ITMS in enhancing muscle strength in athletes compared with IKMS [60], and such result may be related to differences among participants and training volume.

Therefore, for KOA patients, the recruitment of neuromuscular activation may require more attention during the RT process.

This NMA includes numerous conventional therapies such as heat therapy, ultrasound, and manual manipulation. Klemm [61] suggests that thermal stimulation can activate the sensory nervous system, releasing endorphins and other neurotransmitters, thereby reducing pain signals. Additionally, studies indicate that ultrasound also has a thermal effect that can alleviate inflammation and relieve pain [62]. Moreover, Jia [63] believes that ultrasound can directly stimulate cartilage through patella and soft tissues, promoting the preservation of the extracellular matrix, reducing pro-inflammatory mediators, and inducing cell proliferation. Researches show that manual therapy can also activate neural adaptation to reduce pain and improve mobility by loosening adhesions [64, 65]. However, evaluations of manual manipulation vary across different trials, possibly due to differences in the therapists’ skill levels and the types of techniques used. Li [66] notes that although Mulligan and Maitland techniques can improve mobility and relieve joint dysfunction, they are less effective than resistance training in enhancing strength and function. Conventional treatments for KOA and resistance training are not contradictory; they intervene in KOA through different mechanisms to alleviate symptoms.

This NMA encountered several limitations, including the following: grouping entirely based on authors’ descriptions, lack of standards for the execution items, variations in RT grouping training frequencies and volumes and different CG treatment programs. Moreover, the included studies had relatively short observation periods for the outcome indicators, which inhibited the assessment of differences in the potential long-term efficacies of various RTs in the treatment of KOA. Given the relatively small sample size used in this NMA, certain small sample biases may be present, which implies the need for further high-quality clinical trials to verify the efficacy and long-term effect of RT on KOA patients.

## 5 Conclusion

This NMA confirmed the significant benefits of RT in reducing pain, function and knee extension torque in KOA patients, with IKMS being the most effective method. The results of this study may assist clinicians or rehabilitation specialists in the development of appropriate exercise prescriptions.

## Supporting information

S1 Checklist PRISMA NMA checklist of items to include when reporting a systematic review involving a network meta-analysis. (DOCX)

S1 Data (XLSX)

S1 File (DOCX)

S2 File (XLSX)
