# Supplementary material for: Effects of three types of resistance training on knee osteoarthritis: A systematic review and network meta-analysis
Source: PLoS One. 2024 Dec 5;19(12):e0309950. doi: 10.1371/journal.pone.0309950 (PMC11620422; doi:10.1371/journal.pone.0309950)
Supplement: S1 File — (DOCX) [file pone.0309950.s003.docx]

Pubmed

S：

#1 Search:((randomized controlled trial[pt]) OR (controlled clinical trial[pt]) OR (randomized[tiab]) OR (randomly[tiab]) OR (trial[tiab]) OR (groups[tiab])) NOT (animals[mh] NOT humans[mh])

#2 Search:(isometric) OR (isometric contraction) OR (isometric exercise) OR (isometric training) OR (isokinetic) OR (isokinetic contraction) OR (isokinetic exercise) OR (isokinetic training) OR (isotonic) OR (isotonic contraction) OR (isotonic exercise) OR (isotonic training)

#3 Search: Exercise[MeSH]

#4 Search: Resistance Training[MeSH]

#5 Search: Muscle Strength[MeSH]

#6 Search: #2 or #3 or #4 or #5

#7 Search:(knee osteoarthritides) OR (knee osteoarthritis) OR (osteoarthritis of knee) OR (osteoarthritis of the knee)

#8 Search:Osteoarthritis[MeSH]

#9 Search:#7 or #8

#10 Search:#1 and #6 and #9

I

(isometric) OR (isometric contraction) OR (isometric exercise) OR (isometric training) OR (isokinetic) OR (isokinetic contraction) OR (isokinetic exercise) OR (isokinetic training) OR (isotonic) OR (isotonic contraction) OR (isotonic exercise) OR (isotonic training)

Mesh：

isometric contraction

isokinetic contraction

isotonic contraction

Exercise

Resistance Training

Muscle Strength

C：

(knee osteoarthritides) OR (knee osteoarthritis) OR (osteoarthritis of knee) OR (osteoarthritis of the knee)

Mesh

Osteoarthritis


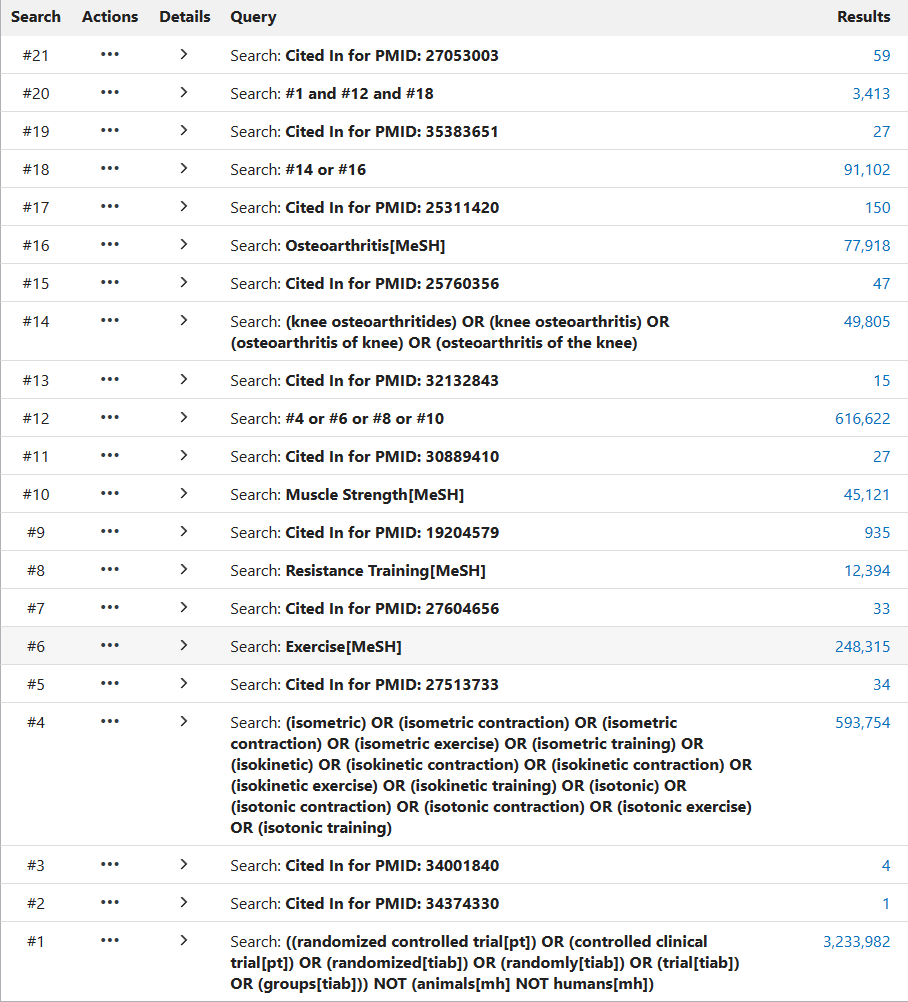


Cochrane Library：

C

(knee osteoarthritides):ti,ab,kw OR (knee osteoarthritis):ti,ab,kw OR (osteoarthritis of knee):ti,ab,kw OR (osteoarthritis of the knee):ti,ab,kw

I：

(isometric):ti,ab,kw OR (isometric contraction):ti,ab,kw OR (isometric exercise):ti,ab,kw OR (isometric training):ti,ab,kw OR (isokinetic):ti,ab,kw OR (isokinetic contraction):ti,ab,kw OR (isokinetic exercise):ti,ab,kw OR (isokinetic training):ti,ab,kw OR (isotonic):ti,ab,kw OR (isotonic contraction):ti,ab,kw OR (isotonic exercise):ti,ab,kw OR (isotonic training):ti,ab,kw

Mesh：

Exercise

Resistance Training

Muscle Strength

isometric contraction

isotonic contraction


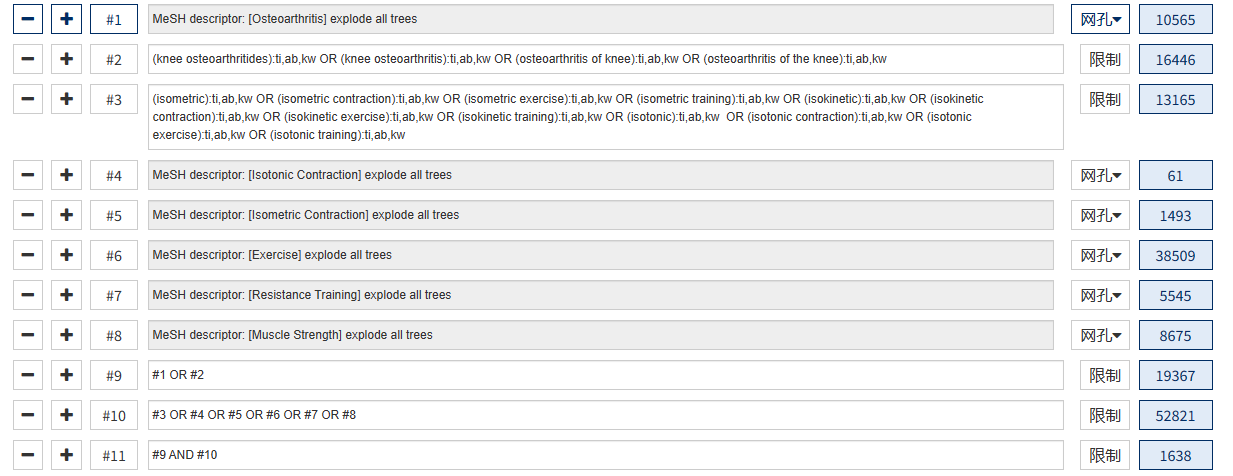


EMBASE

C：

'arthritis, degenerative':ab,ti OR 'arthritis, noninflammatory':ab,ti OR 'arthrosis':ab,ti OR 'degenerative arthritis':ab,ti OR 'degenerative joint disease':ab,ti OR 'noninflammatory arthritis':ab,ti OR 'osteo-arthritis':ab,ti OR 'osteo-arthrosis':ab,ti OR 'osteoarthrosis':ab,ti OR 'primary osteoarthritis':ab,ti OR 'rheumatoid arthrosis':ab,ti OR 'osteoarthritis':ab,ti

Mesh：

isokinetic exercise

muscle isometric contraction

muscle isotonic contraction

I：

'isometric':ab,ti OR 'isometric contraction':ab,ti OR 'isometric exercise':ab,ti OR 'isometric training':ab,ti OR 'isokinetic':ab,ti OR 'isokinetic contraction':ab,ti OR 'isokinetic exercise':ab,ti OR 'isokinetic training':ab,ti OR 'isotonic':ab,ti OR 'isotonic contraction':ab,ti OR 'isotonic exercise':ab,ti OR 'Exercise':ab,ti OR 'resistance training':ab,ti OR 'Muscle Strength':ab,ti
